# Ecthyma gangrenosum in neutropenic patients and the importance of an early skin biopsy for direct examination^⋆^

**DOI:** 10.1016/j.abd.2020.11.018

**Published:** 2022-05-30

**Authors:** Yung Gonzaga, Thiago Jeunon, Jorge Machado, Marcio Nucci

**Affiliations:** a Instituto Nacional de Câncer, Rio de Janeiro, RJ, Brazil; b Private clinic, Rio de Janeiro, RJ, Brazil; c Universidade Federal do Rio de Janeiro, Rio de Janeiro, RJ, Brazil

Dear Editor,

Ecthyma Gangrenosum (EG) was once considered pathognomonic for sepsis caused by *Pseudomonas aeruginosa*.^1^ However, other agents have been described in oncohaematological patients.^2^ Skin biopsies are often not performed, and treatment is usually empirical. This report describes three cases of neutropenic patients with EG who underwent skin biopsy with direct examination.

Patient 1: Male, 36 years old, with acute lymphoblastic leukemia, presented with febrile neutropenia (FN) during chemotherapy, and treatment with cefepime was started. After ten days, he once again had a fever, with the appearance of erythematous lesions with a necrotic center on the face and chest. He had neutropenia (10/mm^3^) and thrombocytopenia (24,000/ mm^3^). Blood cultures and a skin biopsy were collected, and liposomal amphotericin B and voriconazole were started. Direct examination identified septate hyaline hyphae (Fig. 1), which were later also detected in the histopathological examination. *Fusarium spp*. was identified in blood and skin cultures. The patient did not survive depicting of the treatment.

**Figure 1:**
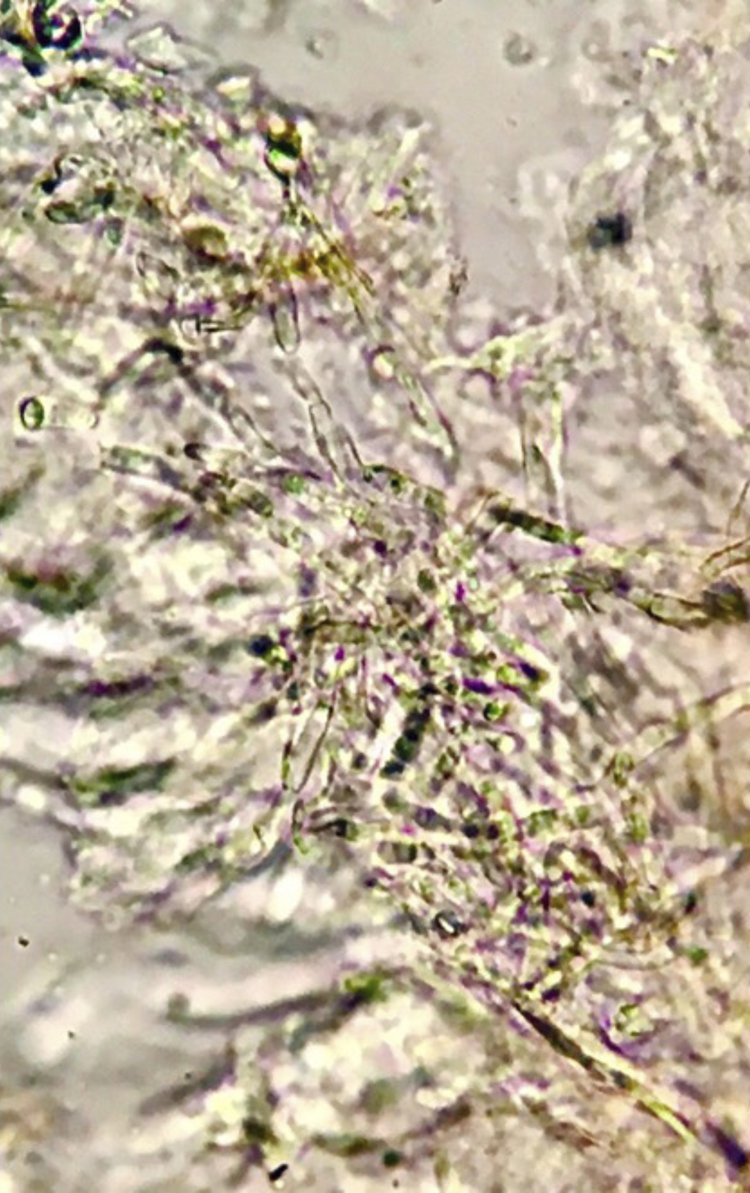
Septate hyaline hyphae with branching at an acute angle. Direct examination, KOH 20%, ×40.

Patient 2: Male, 47 years old, had FN during chemotherapy for acute myeloid leukemia, and treatment with cefepime was started. After fourteen days, he once again had a fever, with the appearance of a single erythematous lesion with a necrotic center, on the chest (Fig. 2). He had neutropenia (40/ mm^3^) and thrombocytopenia (44,000/ mm^3^). Blood cultures and a skin biopsy were collected. Direct examination revealed non-septate hyaline hyphae (Fig. 3), which were later seen in the histopathological examination. Antifungal treatment with liposomal amphotericin B was started. Subsequently, *Syncephalastrum spp*. was identified in the skin culture. The patient remained afebrile, with neutrophilic recovery.

**Figure 2:**
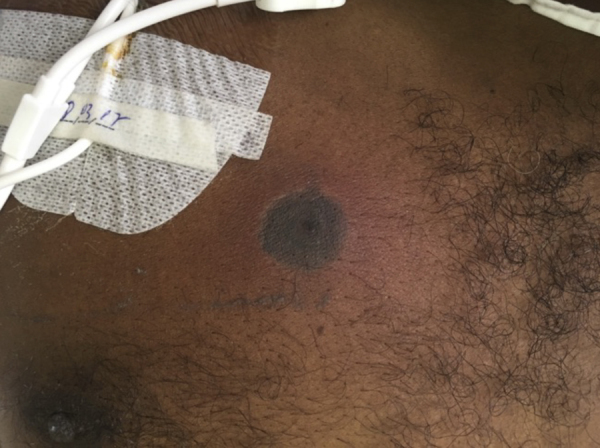
Single erythematous lesion with necrotic center in the anterior thoracic region, close to the Hickman catheter.

**Figure 3:**
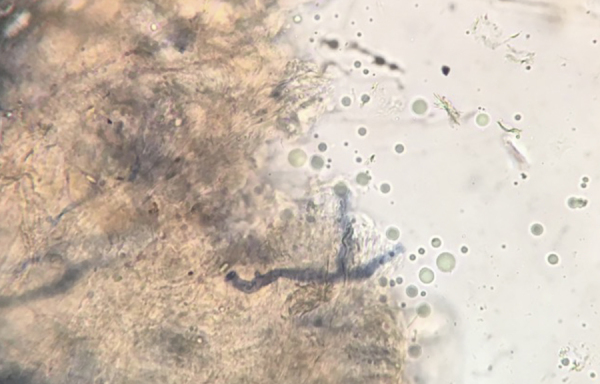
Thick, non-septate hyaline hyphae, branching at a right angle. Direct examination. KOH 20% and Parker's ink.

Patient 3: Male, 36 years old, had FN during chemotherapy for acute myeloid leukemia, and treatment with cefepime was started. After four days, he had a fever, and arterial hypotension, with the appearance of an erythematous lesion with a necrotic center on the back region. Antibiotic therapy was expanded to include polymyxin B, amikacin, and meropenem. He had neutropenia (170/ mm^3^) and thrombocytopenia (2,000/ mm^3^). A skin biopsy was collected, and a direct examination identified the presence of gram-negative rods. No specimen was sent for histopathological examination. Subsequently, multidrug-resistant *Pseudomonas aeruginosa* was identified in the skin and blood cultures. The antimicrobial regimen was maintained until a clinical resolution was attained.

The present report describes three cases of neutropenic patients who developed EG. All had a similar clinical presentation, with fever recurrence and the appearance of lesions during antibiotic therapy. In the first patient, direct examination suggested the diagnosis of fusariosis, directing the combined antifungal treatment. The cause of death was attributed to the absence of immune reconstitution. In the second patient, the direct examination suggested the diagnosis of mucormycosis, directing the change of the antifungal agent for liposomal amphotericin B. In the third patient, the direct examination suggested the diagnosis of bacterial sepsis, directing the expansion of the antibiotic regimen without the association of antifungal agents.

Typically, the EG agent is identified in blood and skin cultures. However, as these results are not promptly available, the treatment is usually the empirical expansion of the anti-infective regimen. The disadvantages of this approach are exposure to toxic drugs and increased costs.

The skin biopsy is a safe procedure in thrombocytopenic patients.^3^ Direct examination helped to identify the agent before the results of cultures were obtained, which later confirmed what had already been detected.

In summary, we suggest performing an early skin biopsy with direct examination in neutropenic patients with EG.

## Financial support

None declared.

## Authors' contributions

Yung Gonzaga: Approval of the final version of the manuscript; critical review of the literature; collection, analysis, and interpretation of data; effective participation in research orientation; intellectual participation in the propaedeutic and/or therapeutic conduct of the studied cases; critical review of the manuscript; drafting and editing of the manuscript; statistical analysis; design and planning of the study.

Thiago Jeunon: Approval of the final version of the manuscript; critical review of the literature; collection, analysis, and interpretation of data; effective participation in research orientation; intellectual participation in the propaedeutic and/or therapeutic conduct of the studied cases; critical review of the manuscript; drafting and editing of the manuscript; statistical analysis; design and planning of the study.

Jorge Machado: Approval of the final version of the manuscript; critical review of the literature; collection, analysis, and interpretation of data; effective participation in research orientation; intellectual participation in the propaedeutic and/or therapeutic conduct of the studied cases; critical review of the manuscript; drafting and editing of the manuscript; statistical analysis; design and planning of the study.

Marcio Nucci: Approval of the final version of the manuscript; critical review of the literature; collection, analysis, and interpretation of data; effective participation in research orientation; intellectual participation in the propaedeutic and/or therapeutic conduct of the studied cases; critical review of the manuscript; drafting and editing of the manuscript; statistical analysis; design and planning of the study.

## Conflicts of interest

None declared.

## References

[1] Pickarrd R., Llamas R. (1970). Ecthyma gangrenosum complicating Pseudomonas bacteremia. Rare survival. J Fla Med Assoc..

[2] Vaiman M., Lazarovitch T., Heller L., Lotan G. (2015). Ecthyma gangrenosum and ecthyma-like lesions: review article. Eur J Clin Microbiol Infec Dis..

[3] Xia F.D., Khosravi H., Waul M.A., Butler D., Joyce C., Mostaghimi A. (2017). Low risk of hemorrhagic complications after obtaining diagnostic skin biopsy specimens in a cohort of thrombocytopenic inpatients. J Am Acad Dermatol..

